# Wearables Meet IoT: Synergistic Personal Area Networks (SPANs)

**DOI:** 10.3390/s19194295

**Published:** 2019-10-03

**Authors:** Emil Jovanov

**Affiliations:** Electrical and Computer Engineering Department, The University of Alabama in Huntsville, Huntsville, AL 35899, USA; emil.jovanov@uah.edu

**Keywords:** wearable monitoring, health monitoring, mHealth, wireless body area networks, IoT, smart stuff, ambient-assisted living, aging in place

## Abstract

Wearable monitoring and mobile health (mHealth) revolutionized healthcare diagnostics and delivery, while the exponential increase of deployed “things” in the Internet of things (IoT) transforms our homes and industries. “Things” with embedded activity and vital sign sensors that we refer to as “smart stuff” can interact with wearable and ambient sensors. A dynamic, ad-hoc personal area network can span multiple domains and facilitate processing in synergistic personal area networks—SPANs. The synergy of information from multiple sensors can provide: (a) New information that cannot be generated from existing data alone, (b) user identification, (c) more robust assessment of physiological signals, and (d) automatic annotation of events/records. In this paper, we present possible new applications of SPANs and results of feasibility studies. Preliminary tests indicate that users interact with smart stuff—in our case, a smart water bottle—dozens of times a day and sufficiently long to collect vital signs of the users. Synergistic processing of sensors from the smartwatch and objects of everyday use may provide user identification and assessment of new parameters that individual sensors could not generate, such as pulse wave velocity (PWV) and blood pressure. As a result, SPANs facilitate seamless monitoring and annotation of vital signs dozens of times per day, every day, every time the smart object is used, without additional setup of sensors and initiation of measurements. SPANs creates a dynamic “opportunistic bubble” for ad-hoc integration with other sensors of interest around the user, wherever they go. Continuous long-term monitoring of user’s activity and vital signs can provide better diagnostic procedures and personalized feedback to motivate a proactive approach to health and wellbeing.

## 1. Introduction

As aging population and their chronical conditions in all developing countries create increasing pressure on healthcare systems, technological solutions and modified healthcare paradigm are crucial [1]. Currently, chronic disease management is mostly *reactive*, while *proactive* approach might deliver much better care at a far cheaper price point. New healthcare paradigms, such as P4 medicine [2], are increasingly accepted as a solution. P4 stands for predictive, preventive, personalized, and participatory medicine. The proposed paradigm provides patients, consumers, and physicians with personalized information for each person’s unique biology and focuses on the causes rather than the symptoms of the disease. By providing personalized feedback, it is possible to motivate active participation and allow users to assess the impact of lifestyle decisions and improve their quality of life. However, this requires massive continuous data collection for the development of the personalized models. 

Technological advances of sensors, wearable health monitors [3], mHealth monitoring [4,5], smart homes, and Internet of Things (IoT) technologies [6] create new opportunities for improving health care and quality of life [7]. In this paper, we present an overview of the field of wearable computing and wearable health monitoring and discuss its relationship to the fields of smart environments and ambient intelligence as a part of IoT systems. We refer to monitors integrated into a person’s environment as ”smart stuff” [8]. Smart stuff provides the context of measurements from wearable monitors and facilitates the synergy of information that generates completely new insights into the state of the user. 

Dynamic integration of personal area networks with smart stuff in our environment creates an ad-hoc network that can facilitate synergistic processing and generate new information. In this paper, we propose the use and present several examples of synergistic personal area networks (SPANs) with support for personalized health monitoring and interventions. We present the general organization, opportunities, and possible applications of SPANs, with results from several preliminary feasibility studies. 

## 2. Wearables

Integration of devices in the personal workspace, such as computers, personal digital assistants, and, later, smartphones led to the development of standards for personal area networks. Personal area networks were first introduced as a term by Zimmerman [9] in 1996 and further developed by the IEEE P802.15 Working Group [10]. We proposed and implemented the first wireless personal area network for health monitoring in 2000 [11]. Development of sensors, wearable computing, and ubiquitous communications by the widespread use of smartphones enabled mobile health, or mHealth [4,12,13]. 

Wearable health monitoring allows monitoring of activities of daily living and; therefore, more accurate diagnostic procedures, unbiased by changes in the hospital environment [3]. The first wearable health monitoring devices were electrocardiogram (ECG) holters, used to detect irregularities in heart rhythm (i.e., arrhythmias). They typically record 3- or 5-lead ECG on a small, battery-operated controller worn on a belt. Sensor electrodes are standard “wet” ECG electrodes attached to the body. After 24–48 h of non-stop use, the holter is returned to a cardiologist for readout and signal processing analysis. The use of standard electrodes limits the total duration of holter use to 3–4 days when standard electrodes typically fall-off. However, some arrhythmias are infrequent and still extremely dangerous and deadly. That requires much longer monitoring, in the order of several months. Therefore, a new generation of implanted ECG recorders, called loop recorders, have been developed [12]. Noninvasive long-term sensing of ECG requires new, innovative methods. 

Wearable sensors and systems must satisfy stringent size and weight requirements. Therefore, they must feature ultralow-power processing and communication (even power scavenging), intelligent on-sensor processing, autonomic sensing, and support data mining and distributed inferencing. 

There are two main facilitators of wearable monitoring: smartphones and smartwatches. Smartphones became the Internet gateway of wearable monitors that support both short-range–low-power communication of wearable sensors and long-range cellular communication as an Internet gateway. That was the foundation of ubiquitous mHealth monitoring [4]. However, smartphones are not always on the body or close enough to the user to maintain connectivity with the wearable sensor, which we refer to as “loose attachment.” Smartwatches resolve that problem with a firm attachment to the user’s hand most of the time. Both devices represent very powerful computing and communication platforms and feature the increasing number of sensors onboard. The first sensors introduced to smartphones were inertial sensors. Although necessary for the general operation of the device, the sensors can be used to monitor the activity of the user [14,15]. Some smartphones feature an optical photoplethysmography (PPG) sensor that can be used to monitor vital signs. However, it requires the positioning of the finger on the sensor. Unlike smartphones, smartwatches are always in contact with the hand. Preliminary studies indicate that their performance is sufficiently good for vital sign monitoring in many health monitoring applications [15,16]. 

One of the main challenges for wearable monitoring is understanding the context of measurements. For example, irregular heart rhythm in the ECG signal can be the result of motion artifacts caused by the movement of electrodes during walking or running. Therefore, understanding the context of measurements improves the quality of measurements, eliminates artifacts, and improves the reliability of measurements and diagnostic procedures. We believe that IoT sensors could provide the context of measurements in many situations. 

## 3. IoT Sensing

Internet of things (IoT) technologies integrate sensing, processing, and communication at an unprecedented scale. IoT-enabled appliances are becoming common in smart homes, with an exponentially growing number of deployed devices. In 2008 the number of things connected to the Internet exceeded the number of people on Earth. It is expected that IoT devices will represent 62% of 29 billion connected devices by 2022, driven by new use cases [17]. 

In addition to smart homes, transportation, and industrial automation, IoT is increasingly used for health applications. Wu et al. integrated wearable sensors for monitoring of the vital signs and the environmental conditions around the subject and an IoT cloud server connected to the Internet [18]. Hassanalieragh et al. show how IoT intelligence can improve the analysis and visualization of health records [19]. Manogan et al. present the IoT patch with edge processing and analysis [20]. Rodrigues et al. present an excellent overview of wearables, ambient sensors, and healthcare solutions that they call the Internet of healthy things (IoHT) [21]. Typical problems include a variety of communication standards (Bluetooth, Bluetooth Low Energy (BLE), Wi-Fi/IEEE 802.11, ZigBee/IEEE 802.15.4, Long Range (LoRa), Near-field Communication (NFC), Radio-frequency identification (RFID), cellphone connectivity), power efficiency of wearable and ambient sensors, seamless sensor discovery and integration, and security and privacy issues and concerns. Implantable sensors provide unique sensing and intervention opportunities (e.g., implanted blood glucose monitoring sensors and insulin pumps). They can be integrated with existing wireless body area networks (WBAN) or communicate in an implantable body area network (IBAN). Darwish et al., presents a recent survey of implantable sensors and systems [22]. 

**Figure 1:**
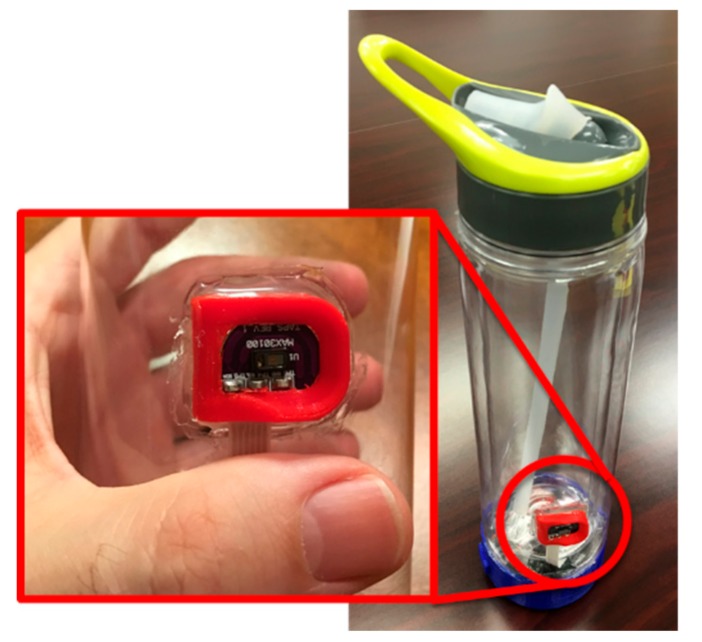
Smart water bottle with embedded vital sign monitor (Smart Stuff) [8].

We implemented a smart water bottle as an IoT appliance, as shown in Figure 1 [8,23]. We selected a water bottle since proper hydration represents one of the most important factors for health and wellbeing. While most users need to increase liquid intake, some user groups, such as heart and kidney patients, have to limit their water intake and still stay properly hydrated. Therefore, we implemented a real-time hydration management platform integrated into a mHealth system that allows users and caregivers access to real-time hydration status and hydration history. We use capacitive sensing to estimate the volume of liquid in the bottle in real-time and report the result periodically, every 60 s in the current version of the controller. The controller communicates with the mHealth server using Wi-Fi or Bluetooth wireless interfaces. To facilitate the monitoring of the activity of patients, the controller integrates a 3D accelerometer and a heart rate monitor. The controller processes 3D acceleration to detect the handling and orientation of the bottle. Our smart water bottle also integrates a custom touch and pulse sensor (see Figure 1). The sensor monitors capacitance to detect the touch of the sensor, which indicates when the bottle is used. Integrated optical PPG sensor MAX30100/30102 [24] is used to monitor the vital signs of the user [25]. 

Capacitive sensing provides a power-efficient measurement of several sensors, such as touch and liquid volume sensor in our smart water bottle. Embedded microcontrollers often support capacitive sensing on multiple pins. We use NXP Kinetis controllers that support capacitive sensing on up to 12 pins [26,27]. We demonstrated that capacitive sensing could be used to directly monitor the heartbeats of users during contact [28]. 

## 4. SPAN: Synergy of Information from Wearables and IoT Sensors

Integration of wireless body area networks with smart environmental sensors and smart objects of everyday use creates new opportunities and applications, particularly for non-invasive continuous health monitoring. 

The most important monitoring functions belong to three groups: vital sign monitoring, activity monitoring, and location sensing.

1. *Vital sign monitoring* provides snapshots or continuous measurements of the user’s vital signs, such as heart rate, respiration rate, and blood pressure.

Vital signs are typically monitored using wearable sensors attached to the human body. We propose the integration of vital sign monitors in objects of everyday use. An example of the smart water bottle designed as IoT appliance with an integrated pulse oximeter and heart-rate controller, or photoplethysmography sensor (PPG), shown in Figure 1. As an alternative, existing signals can be used for so-called opportunistic monitoring; a passive Doppler radar can be used to detect both user activity and breathing [29].

New sensing modalities are being proposed and tested, such as capacitive sensing of vital signs [28], video [30], electromagnetic field monitoring [29], and new sensor materials [31].

2. *Activity monitoring* can be implemented using:
  - Inertial sensors embedded in IoT objects or wearable inertial sensor on user [8];
  - Touch sensors that are typically implemented as capacitive or pressure sensors and can indicate the use of a smart object;
  - Mechanical and magnetic sensors can indicate opening or general use of the device. For example, the smart pill bottle from Adhere Tech can detect the use of the bottle and transmit information in real-time to the medical server to facilitate drug compliance monitoring of patients [32]. Nonadherence in the U.S. is estimated to be $100–$300 billions of avoidable health costs [33].
  - Remote sensing (e.g., video, infrared [34], or electromagnetic field monitoring [29])

3. *Location sensing.* The location of the user could be information itself or provide context for measurements. For example, outdoor location (e.g., home, office, physician’s office, park) provides information about activity during the day, but also the context of measurements, such as:
  - Blood pressure measurements are typically higher when measured in physician’s office than at home;
  - The number of bathroom visits might indicate the development of urinary tract infection;
  - User association for automated measurements, such as assigning automated weight scale measurement with the user closest to the weight scale.

Short-range location sensing can be implemented using wireless RFID tags, but only for up to 15 cm. Longer range solutions include monitoring the strength of wireless signal (received signal strength indicator—RSSI) with limited accuracy, and monitoring of time-of-flight of each transmission that is part of ultra-wide band (UWB) communication at the price of significantly increased power consumption and complexity of devices. 

**Figure 2:**
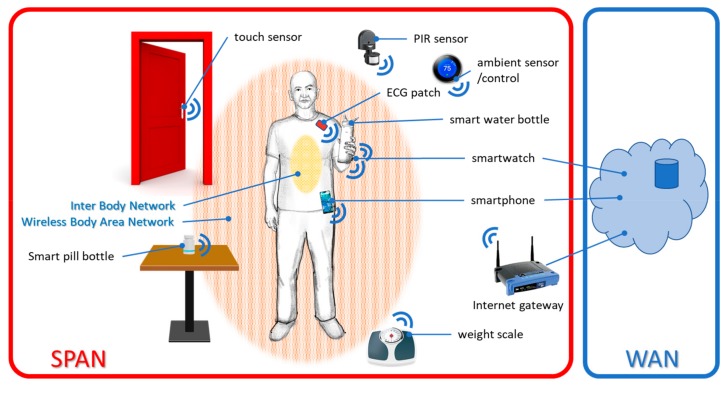
The system architecture of a Synergistic Personal Area Network (SPAN).

The functionality of personal area networks spans multiple domains, as represented in Figure 2:

4. *Inter body network* integrates communication of *Implantables* and *Ingestibles* inside the body, or with a gateway on the body. As an example, Proteus Discover uses an ingestible sensor that communicates with a wearable sensor patch [35,36].
5. *Body area networks/wireless body area networks* integrate sensors on the body and sometimes in the body [37,38].
6. *Wide area networks (WANs)* use wired, long-range wireless, or cell-phone network connections to interface short-range networks to the Internet and store records in the cloud and on the medical server. That allows physicians, users, and their caregivers access to all the records that the user wants to share with them [38].

**Figure 3:**
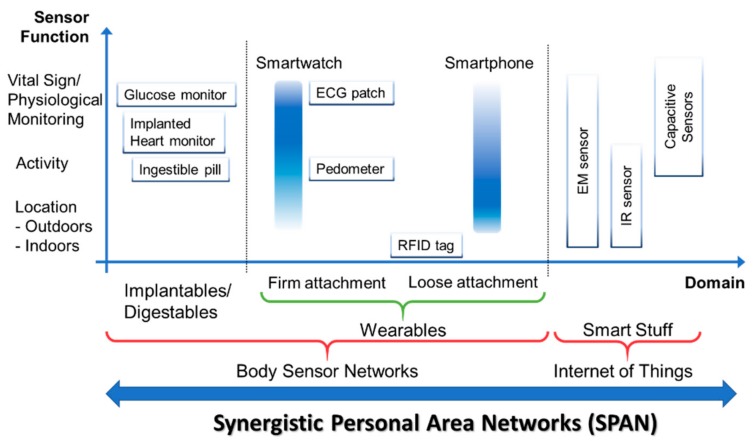
Taxonomy of synergistic personal area networks; ECG—electrocardiogram sensor; IR—infrared sensor.

Figure 3 represents the general taxonomy of networks and functions. Applications span multiple domains and feature application-specific functionality, as described above. 

### 4.1. SPAN Applications

Synergistic processing of information from SPAN sensors creates new opportunities and new applications. We illustrate possible applications of synergistic processing for two classes of applications: (a) user identification, and (b) monitoring of parameters that can be detected only through the synergy of information from multiple sensors. 

#### 4.1.1. User Identification

The synergy of information from wearable and IoT sensors can provide identification of the user that handles the smart IoT object.

**Figure 4:**
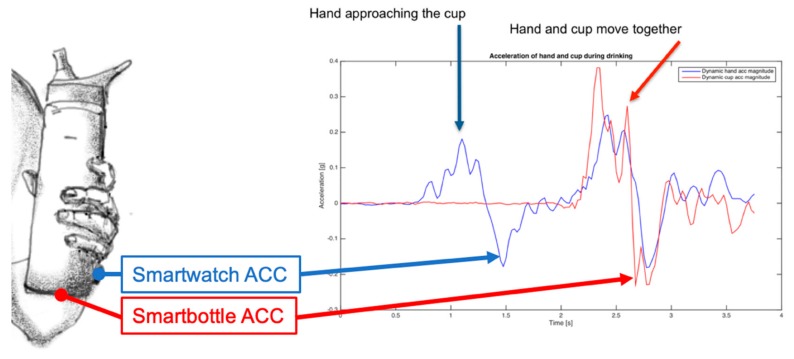
User identification using a pattern of dynamic acceleration (ACC) of the hand and a smart object.

- *Vital sign-based user identification* [39,40]. If the IoT object (smart stuff) contains a vital sign monitor, the user with a wearable sensor that monitors the same vital sign can be identified based on the similarity of vital signs from both sensors. For example, heart rate acquired on the water bottle is compared with heart rate from the smartwatch of users in the vicinity. A similar heart rate or a sequence of heart rate values can identify the subject, particularly in the case of the limited number of subjects sharing the same space (e.g., a couple living together, or nursing home). Subject identification may facilitate the annotation of automatically collected records. Javaid et al. present the use of the wearable ECG and tiles with ballistocardiogram (BCG) for user identification and home monitoring [40].
- *Activity-based user identification*. Interaction with a smart object causes certain activity parameters to be similar, which can lead to user identification. For example, wearable inertial sensors might have some or multiple parameters very similar to the equivalent parameters on the object. We illustrated user identification using the three axis (3D) accelerometer on the smartwatch of the user and in a smart water bottle in Figure 4. A dynamic 3D vector magnitude with no baseline for the smartwatch and the smart water bottle become very similar when the hand holds the water bottle, as can be seen in Figure 4. Therefore, the system can detect if somebody is using my water bottle. That information is critical in nursing homes and hospitals, where detection of the use of a water bottle by an “unauthorized” user might represent a significant health hazard. Moreover, “authorized” users, such as nurses, do not trigger the alarm.
- *Identification of the class of users*, such as child vs. adult. In [41] we present how capacitive sensing on multiple segments of the object can be used to detect a pattern of the contact interface that can be used to detect if the person handling the object is an adult or a child. In the case of the smaller number of known users (e.g., family members), the system can identify individuals using the object. “Smart” bottles equipped with sensing technology have substantial potential to detect hazardous events, provide instant alarms and warnings to children who handle bottles containing dangerous products, and warn parents/guardians, wherever they are, via text message or other means.

#### 4.1.2. Synergistic Physiological Monitoring

The synergy of information collected from multiple sensors may provide crucial new parameters that can be used to seamlessly assess the user’s health state. As an example, we present possible use of two sensors in a SPAN to assess pulse wave velocity (PWV). PWV is the velocity at which blood pressure pulse propagates through the artery. PWV is clinically used to assess the stiffness of arteries with the measurement of the arterial pulse propagation from carotid to femoral artery [42]. However, PWV can be used for continuous and non-invasive assessment of blood pressure [43]. 

**Figure 5:**
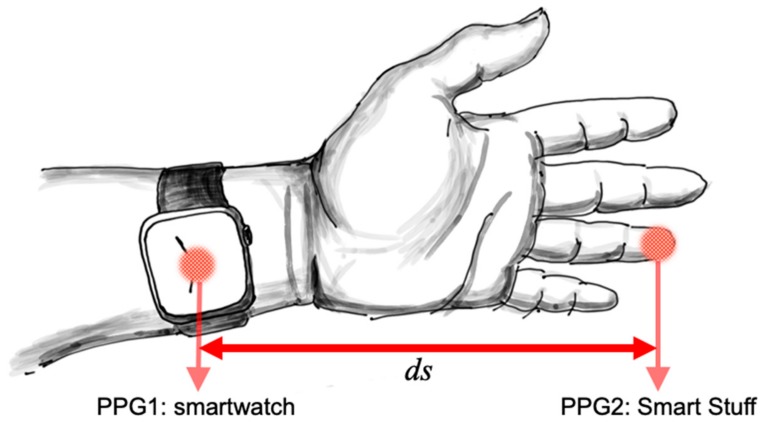
Using the synergy of wearable sensors and IoT objects to assess relative changes in the blood pressure; PPG1 represents photoplethysmogram sensor on the smartwatch and PPG2 is photoplethysmogram sensor on the Smart Stuff.

We propose to use two optical sensors at the known distance, and measure the latency of blood pulse arrival on each sensor, as represented in Figure 5. The first sensor is in the smartwatch (PPG1) and detects the arrival of the blood pulse to the wrist. The second sensor can be on the finger (PPG2) and detects arrival of the blood pulse at the top of the finger. Since the distance between the wrist and the finger is constant (*ds*), if we find latency of the blood pulse/pulse travel time (*dt*) at the finger, we can calculate PWV = *ds*/*dt*. The average carotid-to-femoral PWV of healthy subjects of all ages is 6.84 m/s [44], which means that for the distance between sites of 20 cm, expected time delay is 29 ms. Therefore, the measurement accuracy of 1 ms would generate an error of up to 3.4%. PWV is strongly correlated with age and increases with age (e.g., 9 m/s for the age group over 70 years [44]). Consequently, the same time accuracy would generate an error of 4.5% for subjects over 70 years. PWV depends on the heart rate and breathing, but relative changes of the PWV depend on the current blood pressure of the user [45,46]. All blood vessels can expand or contract as a result of sympathovagal activity; however, since most of the vascular resistance occurs in small blood vessels, changes caused by blood pressure are more visible in the finger PPG [45].

SPANs can integrate and synchronize measurements of PPG from a smartwatch and an object of everyday use equipped with an embedded PPG sensor. Synchronized measurements of two signals, as represented in Figure 5, can be used to calculate the latency of the blood pulse and PWV. An alternative setup would be to use the electrocardiogram (ECG) sensor and PPG sensor on the finger and calculate the time delay between the electrical signal (ECG) and some feature of the optical signal (PPG). The measured time delay is called pulse arrival time (PAT) and can be used for assessment of vascular health [47] and personalized stress level. Another example of synergistic processing is the assessment of blood pressure using ballistocardiography and PPG [48]. Ballistocardiogram can be recorded and processed in a smart weight scale, and PPG on a smartwatch or other wearable sensors. Assessment of blood pressure will be facilitated through the synergy of existing sensors instead of the custom setup of sensors. 

**Figure 6:**
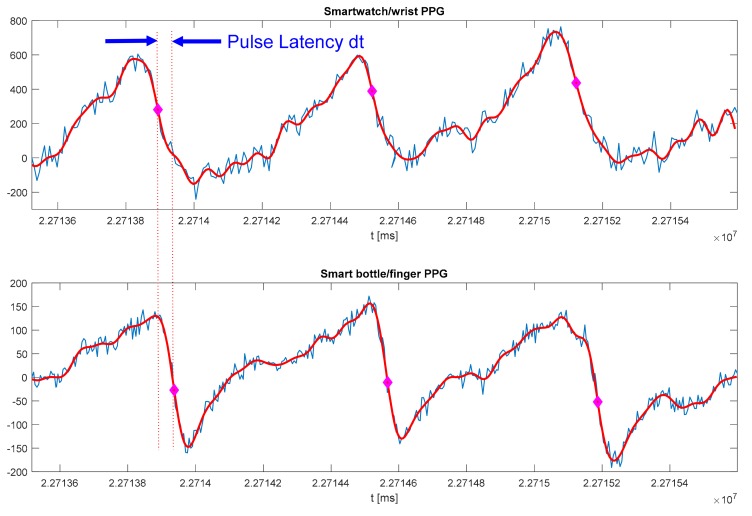
Measurement of the Pulse Wave Velocity (PWV) using smartwatch and PPG sensor embedded in water bottle/object of everyday use (see Figure 5 for measurement setup).

In our preliminary experiment with configuration represented in Figure 5, we used a smart water bottle with a PPG sensor embedded on the outside of the bottle (see Figure 1) and Polar M600 smartwatch. The PPG sensor on the smart bottle resides in the small groove that holds, comfortably, the user’s finger every time the bottle is used (see Figure 1 and [8]). PPG signals recorded on the smartwatch and smart bottle are shown in Figure 6. The bottle controller can communicate with the smartwatch directly, or both sensors can communicate with a custom smartphone application or a home server. SPAN integration using a home server provides “always-on” connectivity, short response times, and precise time synchronization of individual sensors. Precise time synchronization of sensors is crucial for synergistic processing. In our preliminary experiment, we used Raspberry Pi as a home server. Smart bottle controller and smartwatch communicate with the server over Wi-Fi using the Message Queuing Telemetry Transport (MQTT) messaging protocol. The home server sends regular time beacons that are used for time synchronization on individual sensors and receives signals synchronized with the global clock. The application on the home server then filters and processes both signals. Signals are filtered using a low-pass Finite Impulse Response (FIR) filter of order 81, with a cut-off frequency of 15 Hz. We identified individual heartbeats as samples with the maximum negative slope, which is typical for PPG processing. Original PPG, filtered signal, and identified heart beats are shown in Figure 6. More sophisticated processing methods, such as wavelet transform [45], would provide better accuracy and assessment. 

Since the signals from the smartwatch and smart bottle are synchronized, the latency of the PPG on the finger (*dt*) can be found as a time difference between pulse arrival times on the wrist and the finger, as illustrated in Figure 6. We used global timestamps with a resolution of 1 ms starting from midnight (Figure 6). We used a Polar M600 smartwatch because of the quality of the PPG sensor [15] and support for Android Wear OS application development. Android provides time in milliseconds (UNIX format), and sampling event times in nanoseconds, although the actual accuracy of the clock is system dependent. The calculated time delay was then used to assess absolute value and relative change of the pulse wave velocity (PWV). 

In our preliminary experiment, the subject was a 29-year-old male; *ds* was 19 cm (see Figure 5). Measured time delays of the three heartbeats in Figure 6 are 43.17, 44.17, and 39.77 ms that correspond with PWV of 4.4, 4.3, and 4.8 m/s, respectively. Expected PWV for the age group of the subject is 3.92 to 8.14 m/s [44]. A more comprehensive analysis and validation of results will be a subject of the follow-up paper. Therefore, measurement of PWV using synergistic processing from wearable devices (e.g., smartwatch, ECG patch) and smart stuff (e.g., smart water bottle, smart weight scale, smart computer mouse) could provide an assessment of blood pressure. Our preliminary results indicate that synergistic processing in SPANs is feasible. 

The next important question was “how often do we use (potentially) smart objects”? In our pilot usability test, we used 11 subjects, aged 27 to 78 years (mean 45 ± 17.91 years) and asked them to use our automated smart bottle. The average number of bottle interactions was 15.3 times/day, with an average duration of 35 ± 25.4 s [25]. The average number of heartbeats detected during a touch session (individual bottle use) was 34.2 heartbeats or 45.1% of the interaction time. Therefore, the smart water bottle would allow us to seamlessly collect heart rate approximately 15 times per day, sometimes collecting the sufficient number of heartbeats to even assess heart rate variability of the user [25]. Detection of almost half of the heartbeats is an excellent result since clinical measurements of heart rate require a clip to ensure good contact of the finger with the PPG sensor, whereas our smart bottle relies only on the user holding the bottle. 

We encountered several potential issues that can limit applicability of the proposed approach: (a) Motion artifacts can significantly influence quality of PPG; (b) PPG sampling frequency on the smartwatch is low (135 Hz), although sampling frequency on the smart bottle can be more than 1000 Hz; (c) hand position influences measured latency [45]; and d) frequency of smartwatch clocks can drift, which influences synchronization of signals. Our embedded sensor provides much more robust monitoring, since they are specialized only for the monitoring task, while smartwatches also represent a general computing/communication platform. However, we expect that the performance of smartwatches will soon improve. The monitoring community is anxiously waiting for open access to raw PPG signals at a higher sampling rate, and availability of interbeat intervals (IBI). That feature was available on the discontinued Basis smartwatch [16]. 

Our future study will examine how often we get signals that are good enough for synergistic processing. 

## 5. Conclusions and Future Work

Continuous long-term monitoring of user’s activity and vital signs can provide better diagnostic procedures and personalized feedback to motivate a proactive approach to health and wellbeing. Recent advances and exponential use of wearable and IoT technologies provide new opportunities for users and caregivers. “Things” with embedded activity and vital sign sensors that we refer to as “smart stuff” can interact with wearable and ambient sensors. A dynamic, ad-hoc personal area network can span multiple domains and facilitate synergistic processing—SPAN. Synergy of information from multiple sensors can provide novel information that is not available from individual sensors, such as assessment of blood pressure. 

In this paper, we presented possible new applications of SPANs and results of preliminary feasibility studies. Preliminary tests indicate that users interact with smart stuff—in our case, a smart water bottle—dozens of times a day and sufficiently long to collect vital signs of the users. We demonstrated how the synergy of information from wearable devices (e.g., smartwatch) and an object of everyday use (e.g., smart water bottle) could provide user identification and assessment of personalized blood pressure fluctuations every time the object is used. The integration of multiple sensors might even facilitate the assessment of the psychophysiological state, for example, stress level during the day. That allows seamless monitoring and annotation of vital signs dozens of times per day, every day, without the attachment of new sensors or initiation of the measurement procedure. If the system can collect and process individual measurements, we can create personalized monitoring of short- and long-term trends. Collected data sets might provide “big data” necessary for new healthcare paradigms [49]. 

SPANs are not limited to home monitoring; they can provide a dynamic “opportunistic bubble” for ad-hoc integration with other sensors of interest. We might create one synergistic network with smart stuff at home and switch to the second network in our car by integrating sensors in our car’s seat, wheel, etc. [50]. Automatic integration of all records in our medical record can provide more robust monitoring, automatic annotation (e.g., heart rate during driving), and automated user identification (e.g., a seamless update of weight scale measurement). 

Our future work will be focused on pilot tests to evaluate the effectiveness of new systems for health monitoring in the wild and the development of new applications of SPANs. 

## 6. Patents

Several aspects of the proposed concept have been patented or described in the preliminary patent applications. The smart water bottle and the smart stuff concept have been described in patent application Emil Jovanov, “Liquid Container Systems and Methods for Monitoring User Hydration”. Capacitive sensing and human–computer interfaces have been described in preliminary patent application Emil Jovanov, “System and Method of Physiological Monitoring Using Capacitive Sensing”. Smart pill bottle and drug adherence monitoring system have been described in patents U.S. 7,928,835 (April 2011), U.S. 8,754,769 (June 2014), U.S. 9,125,798 (September 2015), U.S. 9,358,183 (June 2016), and U.S. 10,071,023 (September 2018).
